# Integrative Analysis of Transcriptome and Metabolome Sheds Light on Flavonoid Biosynthesis in the Fruiting Body of *Stropharia rugosoannulata*

**DOI:** 10.3390/jof10040254

**Published:** 2024-03-27

**Authors:** Xian Wu, Zhihui Du, Lian Liu, Zhilin Chen, Yurong Li, Shaobin Fu

**Affiliations:** 1 School of Pharmacy, Zunyi Medical University, Zunyi 563000, China; wux0620@sina.com; 2 Guizhou Horticulture Institute, Guizhou Academy of Agricultural Sciences, Guiyang 550009, China; dzh8928088@163.com (Z.D.); liulian632@163.com (L.L.); chenzhilin@126.com (Z.C.)

**Keywords:** flavonoids, *Stropharia rugosoannulata*, fruiting body, metabolomics, transcriptomics

## Abstract

Flavonoids are a diverse family of natural compounds that are widely distributed in plants and play a critical role in plant growth, development, and stress adaptation. In recent years, the biosynthesis of flavonoids in plants has been well-researched, with the successive discovery of key genes driving this process. However, the regulation of flavonoid biosynthesis in fungi remains unclear. *Stropharia rugosoannulata* is an edible mushroom known for its high nutritional and pharmacological value, with flavonoids being one of its main active components. To investigate the flavonoid content of *S. rugosoannulata*, a study was conducted to extract and determine the total flavonoids at four stages: young mushroom (Ym), gill (Gi), maturation (Ma), and parachute-opening (Po). The findings revealed a gradual increase in total flavonoid concentration as the fruiting body developed, with significant variations observed between the Ym, Gi, and Ma stages. Subsequently, we used UPLC-MS/MS and transcriptome sequencing (RNA-seq) to quantify the flavonoids and identify regulatory genes of Ym, Gi, and Ma. In total, 53 flavonoid-related metabolites and 6726 differentially expressed genes (DEGs) were identified. Through KEGG pathway enrichment analysis, we identified 59 structural genes encoding flavonoid biosynthesis-related enzymes, most of which were up-regulated during the development of the fruiting body, consistent with the accumulation of flavonoids. This research led to the establishment of a comprehensive transcriptional metabolic regulatory network encompassing flavonoids, flavonoid synthases, and transcription factors (TFs). This represents the first systematic exploration of the molecular mechanism of flavonoids in the fruiting of fungi, offering a foundation for further research on flavonoid mechanisms and the breeding of high-quality *S. rugosoannulata*.

## 1. Introduction

The diverse family of flavonoids, widely distributed in plants, plays a crucial role in their growth, development, and stress adaptation [1,2]. They contribute to the main active components of natural medicinal plants, exhibiting various pharmacological functions such as antioxidant [3,4], antiviral, anticancer [5], antiallergic, and anti-inflammatory effects [6].

Flavonoids are crucial secondary metabolites synthesized through the phenylpropanoid and flavonoid biosynthesis pathways [7,8]. The process begins with the conversion of L-phenylalanine into 4-coumarate CoA, catalyzed by *PAL*, *C4H*, and *4CL*, enzymes in the upstream phenylpropanoid pathway. *CHS* then utilizes one p-coumaroyl CoA molecule and three malonyl CoA molecules to form naringenin chalcone, subsequently isomerized to (2S)-Naringenin, a crucial precursor for various flavonoids, via *CHI*. Following this, biochemical reactions catalyzed by *FNS*, *IFR*, or *F3H* lead to the production of flavones, isoflavones, or dihydroflavonols, respectively. Dihydroflavonols are then converted to flavonols by *FLS*, or by *DFR* and *ANS* to anthocyanidins [9,10].

Intensive research on flavonoid biosynthesis in plants has led to the successive discovery of key genes [11]. However, the regulation of flavonoid biosynthesis in fungi remains unclear. Unlike plants, mushrooms lack genes encoding most enzymes involved in the flavonoid synthesis pathway. For example, only three relevant enzymes were annotated from *Auricularia cornea* [12], and despite the detection of 81 flavonoids in *Sanghuangporus baumii*, only four enzymes involved in flavonoid biosynthesis were identified [13]. Notably, these reports utilized mycelium as the experimental material, leaving the process in the fruiting body unaddressed [14]. Studies have found that gene expression differences among mycelium and fruiting body will lead to some differences in the metabolites [15,16].

*Stropharia rugosoannulata* Farl. Ex Murrill is widely cultivated and currently considered one of the top ten commercial mushrooms [17]. This edible and medicinal mushroom is highly valued for its nutritional and pharmacological properties [18]. It is also recommended by the FAO as suitable for developing countries. Furthermore, *S. rugosoannulata* exhibits various pharmacological effects such as antioxidant, bacteriostatic, antiviral, antitumor, and hypoglycemic activities [19,20,21,22]. Its main active components include flavonoids, polysaccharides, and triterpenoids [23,24,25]. Previous studies have focused on the extraction technology and pharmacological activity of total flavonoids from *S. rugosoannulata*, but the specific flavonoids that can be synthesized from *S. rugosoannulata* and how they are synthesized remain unknown.

This study aimed to examine the dynamic characteristics of total flavonoid content and its relationship to fruiting body development stages in *S. rugosoannulata*. Total flavonoids were extracted and determined at four stages: young mushroom (Ym), gill (Gi), maturation (Ma), and parachute-opening (Po). To examine the classes of flavonoids synthesized and their mechanisms in mushrooms, we used widely targeted metabolomics and RNA-seq to analyze a series of fruiting bodies at different time points. Comparative analysis was then performed to identify differentially accumulated flavonoids (DAFs) and differentially expressed genes (DEGs). Finally, a deeper understanding of flavonoid properties and candidate regulatory genes in *S. rugosoannulata* was achieved through the integration of metabolites and gene data. The aim of this study was to explore the flavonoid synthesis pathway in the fruiting body of *S. rugosoannulata* and provide a basis for its utilization of this mushroom in the production of flavonoids.

## 2. Materials and Methods

### 2.1. S. rugosoannulata Cultivation and Material Collection at Different Stages

We obtained the *S. rugosoannulata* strain “SA02” (GenBank accession number SRR18808153) from the Guizhou Academy of Agricultural Sciences. Initially, we cultured the strain on potato dextrose agar (PDA) plates at 25 °C under dark conditions for 7 days. Then, we punched out agar plugs and inoculated them in corn meal liquid medium (3% corn meal, 2% sucrose, 0.2% peptone, 0.1% yeast extract) on a rotary shaker incubator at 160 r/min at 25 °C for 8 days. Subsequently, the mycelia and liquid were transferred to a solid medium containing 80% corncob, 10% wheat bran, 7% corn meal, 2% sucrose, and 1% calcium carbonate. After thoroughly mixing, we added the substrate to polypropylene cultivation bags, each containing an average of 1200 g with a moisture content of 65–70%. The bags were sterilized at 121 °C for 120 min and then incubated at 25 °C under dark conditions until the mycelia completely covered the cultivation substrate.

**Figure 1:**
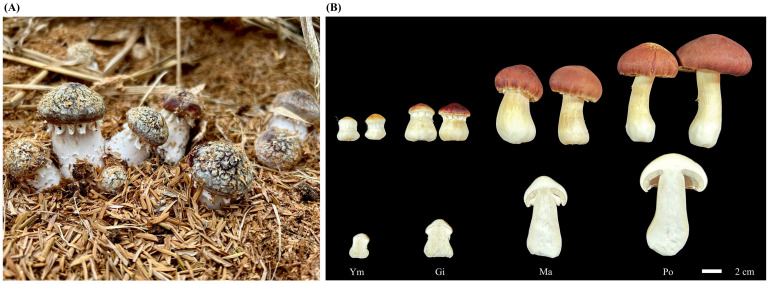
Representative images of *S. rugosoannulata* fruiting body development. (**A**) Fruiting body of *S. rugosoannulata* growing in its controlled environment. (**B**) Samples collected at different developmental stages for experimental research: Ym, young mushroom stage; Gi, gill stage; Ma, maturation stage; Po, parachute-opening stage.

Next, we inoculated pure culture *S. rugosoannulata* mycelium into a cultivation substrate composed of wood chips and brown chaff in a 1:10 ratio. We maintained regular watering during mushroom incubation. We then collected all samples from four fruiting body stages at the same location and time point and immediately froze them in liquid nitrogen, followed by storage at −80 °C. Each stage comprised nine biological replicates, and each analysis, including total flavonoid determination, flavonoid metabolomics determination, and RNA extraction, was performed in triplicate (Figure 1).

### 2.2. Extraction and Analysis of Total Flavonoids

To determine the total flavonoid content in the samples, the sodium nitrite–aluminum nitrate colorimetric method was used [26]. Initially, the samples were ground and mixed evenly. Following this, 0.3 g of the sample was precisely weighed and subjected to extraction through reflux with 60% ethanol for two cycles. The filtrates were combined and evaporated to dryness and then dissolved with 60% ethanol before being transferred to a 25 mL flask and made to a constant volume. This process yielded the extract liquids from four stages of the fruiting body of *S. rugosoannulata*. Subsequently, rutin reference solutions ranging from 0 to 1.2 mL were precisely measured and placed into 10 mL volumetric flasks, with 2 mL of 60% ethanol added to each. After shaking them evenly and allowing them to stand for 6 min, 0.5 mL of 5% NaNO_2_ solution and 10% Al(NO_3_)_3_ solution were successively added. The mixture was then further treated with 4 mL of 4% NaOH solution, and the volume was fixed to 10 mL with 60% ethanol. It was shaken evenly and left to stand for approximately 15 min. The absorbance at the wavelength 510 nm was determined using the corresponding reagent solutions as the blank reference, with the concentration of the reference substance plotted on the X-axis and the absorbance on the Y-axis to draw a standard curve.

Analyses of twelve samples from four periods were performed in triplicate. Significant analyses of the four period samples were calculated using *t*-test analysis by the IBM SPSS statistics 24.0. The average data were displayed plus or minus the standard deviation (average ± SD).

### 2.3. Widely Targeted Metabolomics Analysis of Flavonoid Metabolites in Fruiting Body of S. rugosoannulata

UPLC–MS/MS (ExionLC™ AD; Tandem mass spectrometry, Applied Biosystems 4500 QTRAP, Waltham, MA, USA) was used for qualitative and relative quantitative analysis of the flavonoids in the fruiting body of *S. rugosoannulata*. The samples were first vacuum freeze-dried using a lyophilizer (Scientz-100F, Ningbo, China) and then ground into a powder at 30 Hz for 1.5 min using a grinder (MM 400, Retsch, Haan, Germany). Following this, 50 mg of the sample powder was precisely weighed and 1200 μL of −20 °C pre-cooled 70% methanolic aqueous internal standard extract was added. Next, the mixture was centrifuged at 12,000 rpm for 3 min, and the resulting supernatant was aspirated and filtered through a 0.22 μm microporous membrane to prepare it for UPLC-MS/MS analysis. The UPLC-MS/MS assay strategy employed was based on the research published by the Guizhou Academy of Agricultural Sciences [25].

The quantization of flavonoid metabolites involved the use of multi-reaction monitoring mode (MRM) and a triple quadrupole mass spectrometry. Flavonoids were identified according to chromatographic retention time, molecular weight (*m*/*z*), and MS/MS fragmentation patterns of the standards in a database of MetWare Biotechnology Co., Ltd. (Wuhan, China). The resulting mass spectrometry data were then processed using the Analyst 1.6.3 software. To ensure the accuracy of the quantitative data, methanol was utilized as the internal standard. Unsupervised principal component analysis (PCA) was conducted using the prcomp function in rStudio v4.3.1 after unit variance scaling. Furthermore, the variable importance of the projection (VIP) score of the OPLS-DA model was employed to identify global metabolic changes within the subgroup of samples [27]. Differential metabolites were identified based on a VIP score > 1 and an absolute log2 (fold change) ≥ 1.0.

### 2.4. Gene Expression Profile Sequencing and Transcriptome Data Analysis

Total RNA of the samples was extracted using Trizol (Invitrogen, Waltham, MA, USA), followed by monitoring of RNA degradation and contamination on 1% agarose gels. Afterwards, the quality of the RNA was checked and measured using the Nano Photometer^®^ spectrophotometer (IMPLEN, Westlake Village, CA, USA) and the Qubit^®^ RNA Assay Kit in Qubit^®^2.0 Fluorometer (Life Technologies, Carlsbad, CA, USA). RNA integrity was then assessed using the RNA Nano 6000 Assay Kit of the Bioanalyzer 2100 system (Agilent Technologies, Santa Clara, CA, USA). Sequencing libraries were generated using the NEBNext^®^ UltraTM RNA Library Prep Kit for Illumina^®^ (NEB, Ipswich, MA, USA), following the manufacturer’s recommendations, with index codes added to attribute sequences to each sample. Library fragments were purified using the AMPure XP system (Beckman Coulter, Beverly, MA, USA) to select cDNA fragments of preferentially 250~300 bp in length, and then PCR amplification was performed with Phusion High-Fidelity DNA polymerase. At last, RNA-Seq was carried out on the Illumina Novaseq platform (San Diego, CA, USA) to generate 150 bp paired-end reads.

Raw data processing with Fastp involved trimming sequencing adapters and filtering low-quality reads for improved data quality and reliability. Specifically, paired reads with an N base exceeding 10% of the reads and containing a number of low-quality (Q ≤ 20) bases constituting more than 50% of the entire read length were removed [28]. Following data filtering, transcriptome assembly was conducted using Trinity v2.13.2, and CDS prediction was performed using Trans Decoder v5.3.0 to obtain high-quality clean reads. Subsequently, the transcripts underwent clustering and redundancy removal using Corset v1.09. Finally, the assembled transcripts were compared against various public databases, including NR (NCBI non-redundant protein sequences), Swiss-Prot, Pfam (Protein family), COG, KOG (euKaryotic Ortholog Groups), GO (Gene Ontology), and KEGG (Kyoto Encyclopedia of Genes and Genomes), to obtain functional annotation information for the transcripts.

RSEM was utilized to estimate gene expression levels, followed by normalization of each transcript’s gene expression using the fragments per kb of transcript per million (FPKM) method [29]. To identify differentially expressed genes (DEGs) between sample groups, we used DESeq2 v1.22.2 for differential expression analysis. The corrected *p*-value (FDR < 0.05) and |log2 (fold change)| ≥ 1 were used as the threshold for significant difference expression after correcting using the Benjamini & Hochberg method [30]. DEGs were, respectively, mapped to each pathway of the KEGG database (http://www.genome.jp/kegg/ accessed on 23 August 2023), the number of genes in each pathway was counted and then clusterProfiler v 4.6.0 was used to analyze the statistical enrichment of DEGs in the KEGG pathways [31].

### 2.5. Combined Analysis of Flavonoid Metabolites and Transcriptomic

The FPKM values of DEGs were normalized with the R scale function for K-means clustering. Moreover, for a comprehensive understanding of the relationship between genes and metabolites, Pearson correlation coefficients were computed for DEGs involved in flavonoid biosynthesis and flavonoid metabolites. Correlations with a coefficient absolute value exceeding 0.9 were considered significant. Ultimately, the correlation network was visualized using Cytoscape software (Version number 3.9.1) [32].

### 2.6. Quantitative Real-Time PCR Analysis

Twelve genes involved in flavonoid biosynthesis with three replicates each were chosen for quantitative real-time fluorescent PCR (qRT-PCR) assays to validate the reliability of transcriptome results. First-strand cDNA was synthesized from RNA samples using HiScript^®^ III RT SuperMix kit with a gDNA eraser (Vazyme, Nanjing, China). In addition, ChamQ Universal SYBR qPCR Master Mix (Vazyme, Nanjing, China) was used as a premix for the qPCR reaction, and qRT-PCR was conducted on a CFX system (Bio-Rad, Shanghai, China). Relative expression levels were calculated using the 2^−ΔΔCt^ method with 18S rRNA as the internal control. Primer sequences for qRT-PCR are provided in Supplementary Table S1 [33].

## 3. Results

### 3.1. Measurement of Total Flavonoid Content in Fruiting Body of S. rugosoannulata at Developed Periods

**Figure 2:**
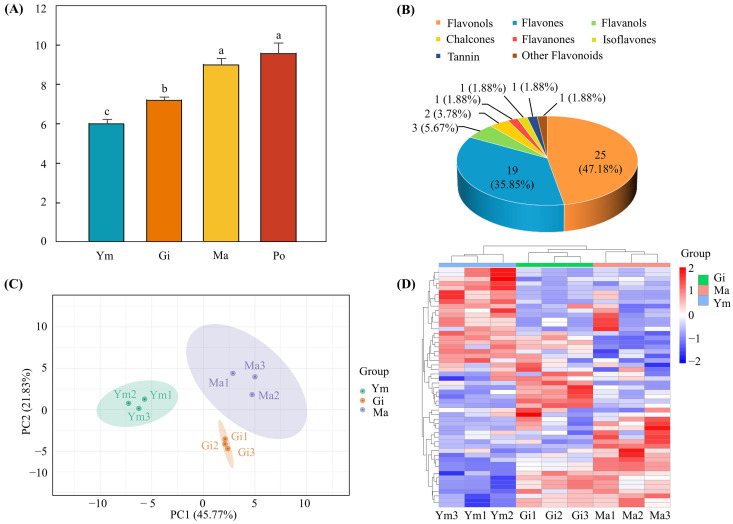
(**A**) The total flavonoid content in fruiting body of *S. rugosoannulata* at four stages. Error bars represent standard deviation (SD). Lowercase letters denote significant differences (*p* < 0.05) between group means. (**B**) Classification and relative abundance of 53 flavonoid metabolites in the fruiting body of *S. rugosoannulata*. PCA (**C**) and hierarchical clustering heatmap (**D**) of flavonoids in nine different samples from the fruiting body of *S. rugosoannulata*.

To explore the dynamic changes of flavonoids in the developmental stages of the fruiting body of *S. rugosoannulata*, the total flavonoid content at the Ym, Gi, Ma, and Po stages was extracted and determined. The results showed that the total flavonoid content in Ym (6.046 ± 0.195 mg/g, FW) was lower than that in Gi (7.231 ± 0.143 mg/g, FW) and Ma (9.030 ± 0.303 mg/g, FW), and it reached its peak at Po (9.609 ± 0.506 mg/g, FW), indicating that the total flavonoid content gradually increased as the fruiting body expanded. Furthermore, variance analyses revealed significant differences in the total flavonoid content between Ym, Gi, and Ma, with a trend of increasing content, but no significant difference was observed at the Po stage (Figure 2A). Therefore, we selected samples in the Ym, Gi, and Ma stages for subsequent metabolome and transcriptome analyses to investigate flavonoid biosynthesis in the fruiting body of *S. rugosoannulata*.

A total of fifty-three flavonoid metabolites, including twenty-five flavonols, nineteen flavones, three flavanols, two chalcones, one flavanone, one isoflavone, one tannin, and one other flavonoid, were identified in the fruiting body of *S. rugosoannulata* during three developmental stages by UPLC-MS/MS (Figure 2B, Table S2). To ensure the reproducibility of the samples under the same treatment, quality control (QC) samples were prepared from a mixture of sample extracts. The overlapped TIC plots of different QC samples revealed consistent peak times and peak heights of the same metabolites, indicating high data reliability and supporting the use of the metabolome data (Figure S1).

PCA analysis was used to characterize the global metabolomics differences among groups and variability between the intra-group samples. The PCA score plot divided the samples into three groups, showing clear separation of flavonoid components in the fruiting body of *S. rugosoannulata* and clustering in replicates, highlighting significant differences in flavonoid metabolites between Ym, Gi, and Ma. Meanwhile, the scores plot indicated a clear separation of Ym from Gi and Ma, suggesting evident differences in flavonoid metabolites in Ym compared to Gi and Ma (Figure 2C). These results were further supported by the heatmap of hierarchical cluster analysis, which confirmed the presence of differences among the flavonoid metabolites of the three sample groups (Figure 2D).

### 3.2. Different Accumulation of Flavonoids in Three Stages

The differential accumulation of flavonoids in the fruiting body of *S. rugosoannulata* at three different developing periods was further compared using OPLS-DA pairwise comparison analysis of flavonoids in Ym, Gi, and Ma. The quality of the OPLS-DA model was assessed using the “overfitting” test, which revealed R^2^Y and Q^2^ values exceeding 0.9 for each model, indicating strong discriminatory ability for the study samples (Figure S2).

**Figure 3:**
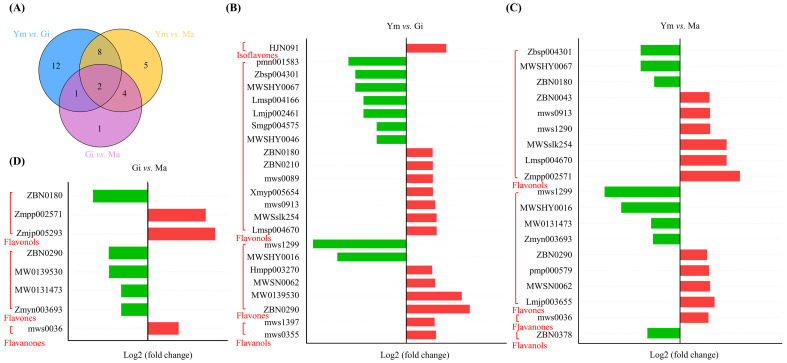
(**A**) Classification and proportion of DAFs in three stages. (**B**–**D**) DAFs between different comparison groups, red for up-regulated and green for down-regulated.

The differential accumulation of flavonoids (DAFs) was assessed using the criteria |log2 (fold change)| ≥ 1 and VIP > 1. As shown in Figure 3A, a total of 33 DAFs were identified across all pairwise comparisons, with flavonols (18, 54.55%) being the predominant category, followed by flavones (10, 30.30%). Notably, kaempferol-3-O-(4″-O-acetyl)rhamnoside and acacetin-7-O-glucoside exhibited differential levels in all pairwise comparisons, indicating their sensitivity to the growth process of *S. rugosoannulata*. Furthermore, the highest number of DAFs (23) was observed in the Ym vs. Gi comparison, with 14 up-regulated and 9 down-regulated compounds. Ym vs. Ma revealed eleven increased and eight decreased flavonoid compounds, while Gi vs. Ma showed only eight DAFs, suggesting a similarity in flavonoid components between the Gi and Ma stages (Figure 3, Table 1). This suggests that the accumulation of flavonoid compounds in *S. rugosoannulata* occurs mainly during the early growth stages of its fruiting body. Moreover, the increased levels of flavones and flavonols primarily contribute to the overall rise in total flavonoid content (Table S3).

### 3.3. Transcriptome Analysis of S. rugosoannulata during Three Periods

Nine cDNA libraries, constructed from the same samples used for metabolic analysis, were submitted to RNA-seq analysis to investigate the potential molecular mechanisms underlying flavonoid synthesis in the fruiting body of *S. rugosoannulata* during three different developing stages. A total of 564,586,690 raw reads and 549,381,244 clean reads were generated across the nine samples, with a Q30 percentage above 94% and an average GC content of 52.53%, attesting to the high quality and quantity of data obtained through transcriptome sequencing (Table S4). After that, all clean reads were de novo assembled using Trinity to generate transcript assemblies and further annotated through the NR, Pfam, Swiss-Prot, COG, GO, KOG, and KEGG databases with an e-value threshold of 1 × 10^−5^, resulting in 49,277 transcripts and 36,096 unigenes.

**Figure 4:**
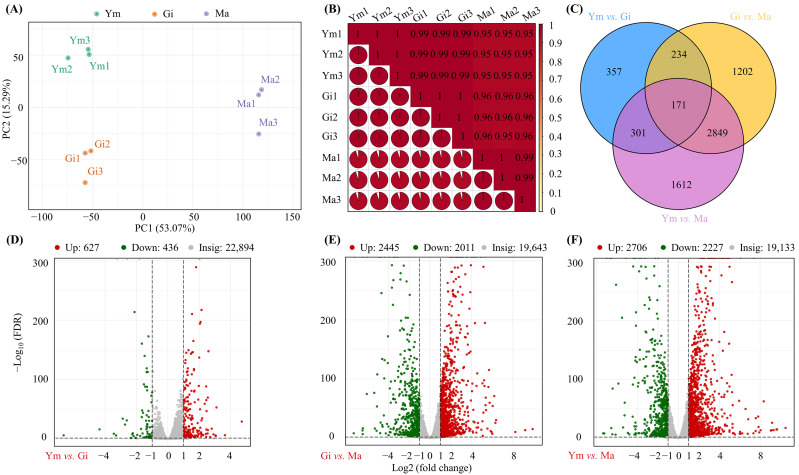
(**A**,**B**) PCA analysis of gene expression among Ym, Gi, and Ma and Pearson correlation coefficients. (**C**) Classification and relative proportion of differentially expressed genes (DEGs) in three stages. (**D**–**F**) Volcano plots of differentially expressed genes (DEGs) between (**D**) Ym vs. Gi (**E**) Gi vs. Ma, (**F**) Ym vs. Ma.

PCA analysis was performed to examine variation in gene expression among the Ym, Gi, and Ma stages. Figure 4A demonstrates distinct clustering of samples from the same growth period, while also showing clear separation between different growth stages. Meanwhile, Pearson correlation analysis showed high absolute values of r (close to 1) between replicate samples (Figure 4B). These results confirm that the data were suitable for subsequent in-depth analysis.

Using FPKM data, we identified a total of 6726 differentially expressed genes (DEGs) among fruiting body samples (Figure 4C). Of these, 171 genes were consistently differentially expressed across all comparisons. Specifically, we observed 1063 DEGs (627 up-regulated and 436 down-regulated) in Ym vs. Gi, 4456 DEGs (2445 up-regulated and 2011 down-regulated) in Gi vs. Ma, and 4933 DEGs (2706 up-regulated and 2227 down-regulated) in Ym vs. Ma (Figure 4, Table S5). It could be seen that the number of DEGs between pairs of groups gradually increased as the fruiting bodies gradually matured.

To identify the flavonoid-related pathways associated with the DEGs, we mapped them to the KEGG database. The analysis revealed the main pathways linked to flavonoid metabolism during *S. rugosoannulata’s* growth to be phenylpropanoid biosynthesis (ko00940), flavonoid biosynthesis (ko00941), isoflavonoid biosynthesis (ko00943), and flavone and flavonol biosynthesis (ko00944).

**Figure 5:**
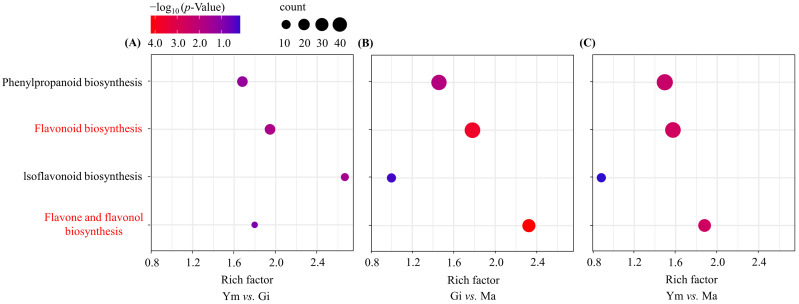
Enrichment analysis of DEGs involved in flavonoid biosynthesis pathway across various sample pairs. (**A**) Ym vs. Gi; (**B**) Gi vs. Ma; (**C**) Ym vs. Ma. Count represents the total number of genes related to the enriched pathway. The rich factor was calculated as the ratio of the number of genes in the pathway entry in the differentially expressed gene to the total number of genes in the pathway entry in all the annotated genes, which indicates the degree of pathway enrichment. Pathways with significant enrichment are shown in red.

The phenylpropanoid biosynthesis pathway progressively increased in enrichment, reaching its highest level in the Ym vs. Ma comparison (Figure 5). The flavonoid biosynthesis pathway was significantly enriched in all comparisons, indicating its crucial role in flavonoid accumulation in the fruiting bodies of *S. rugosoannulata*.

In contrast, the isoflavonoid biosynthesis pathway showed significant enrichment in the Ym vs. Gi comparison but decreased in both Gi vs. Ma and Ym vs. Ma, suggesting potential isoflavone accumulation during the early growth period.

Interestingly, the enrichment of the flavone and flavonol biosynthesis pathway was comparatively low in the Ym vs. Gi comparison, but it rapidly increased and reached its peak in the Gi vs. Ma comparison as *S. rugosoannulata* matured, aligning with the rising total flavonoid levels in the fruiting body (Table 2).

### 3.4. Genes Involved in Flavonoid-Related Pathways in the Fruiting Body of S. rugosoannulata

This study constructed a profile of the flavonoid biosynthesis pathway in the fruiting body of *S. rugosoannulata* by identifying 59 structural genes encoding flavonoid biosynthesis-related enzymes through KEGG pathway enrichment (Table S6). We then examined the expression patterns of key genes and metabolites to understand the pathway more deeply.

**Figure 6:**
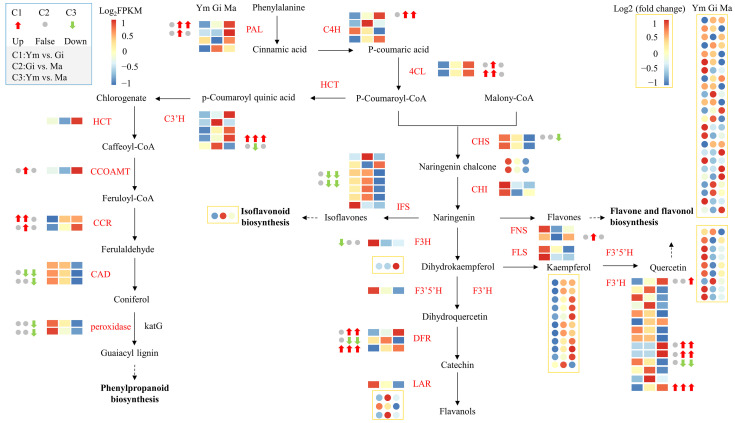
The gene expression profiles of enzymes involved in flavonoid biosynthesis and flavonoid metabolites in the fruiting body of *S. rugosoannulata* at three different stages (Ym, Gi, Ma). Squares indicate genes, circles indicate flavonoid metabolites. Pathways are represented by dotted lines. Abbreviations are shown in Table S6.

Figure 6 demonstrates that the expression of structural genes encoding *PAL*, *C4H*, and *4CL* in the phenylpropanoid biosynthesis pathway, the upstream pathway for flavonoid synthesis, gradually increases with the growth of the fruiting body. This suggests that these genes play a crucial role in regulating flavonoid synthesis in *S. rugosoannulata*. Additionally, we annotated 14 structural genes encoding enzymes in the downstream branches of the phenylpropanoid pathway. These genes might be involved in other synthetic pathways, providing precursors for flavonoid synthesis [34].

Interestingly, most of the remaining structural genes involved in downstream flavonoid biosynthesis were more active in the Ym and Gi stages, possibly contributing to the significant difference in flavonoid metabolites between the young and gill stage mushrooms.

Our analysis identified 14 structural genes encoding *F3*′*H*, and their expression patterns mostly mirrored the accumulation pattern of quercetin. Therefore, we concluded that these *F3*′*H* genes are primarily involved in the flavone and flavonol biosynthesis pathway, which likely contributes to the higher abundance of flavones and flavonols in the fruiting body of *S. rugosoannulata* compared to other flavonoid groups.

We observed significant differences in gene expression between all comparisons (Table 3). Notably, *F3*′*H14* and *DFR3* exhibited significant differences in expression levels across all comparisons. Additionally, the expression levels of *PAL1*, *C4H1*, *4CL2*, *F3*′*H9*, *F3*′*H10*, and *DFR1* show a significant increase during the later stages of fruiting body growth. Therefore, these eight structural genes play a crucial role in the accumulation of flavonoids in *S. rugosoannulata*.

### 3.5. Conjoint Analysis of Transcriptome and Metabolome Profile

**Figure 7:**
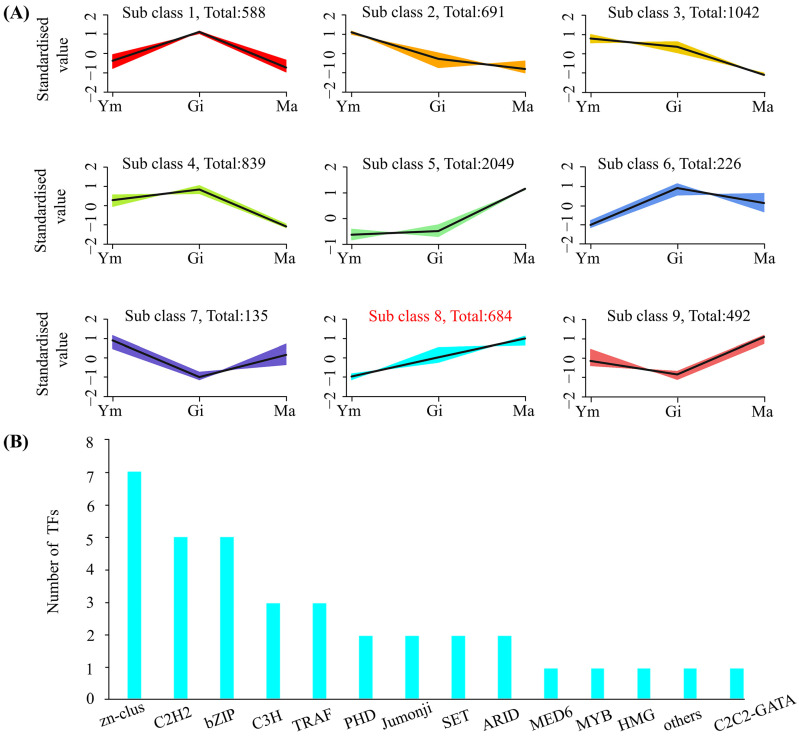
(**A**) K-means clustering of co-expressed genes and their expression patterns. (**B**) Transcriptome factors (TFs) involved in cluster 8.

Following normalization of FPKM values in *S. rugosoannulata* fruiting bodies, a K-means clustering analysis was conducted to explore potential transcriptional regulatory relationships between flavonoid metabolites and genes. The analysis grouped the 6726 DEGs into nine clusters based on similar molecular functions (Figure 7A). Interestingly, one cluster, containing 684 DEGs, exhibited an expression trend consistent with the accumulation pattern of total flavonoids. Closer examination of this cluster revealed the presence of ten differentially expressed genes involved in flavonoid biosynthesis (two *4CL*, two *F3*′*H*, two *CCR*, two *C3*′*H*, one *DFR*, and one *C4H*), potentially playing a regulatory role in flavonoid synthesis. Moreover, the cluster contained 36 transcription factors (TFs), including *C3H*, *C2H2*, *TRAF*, and *MYB*, suggesting their potential involvement in regulating flavonoid biosynthesis in *S. rugosoannulata*.

**Figure 8:**
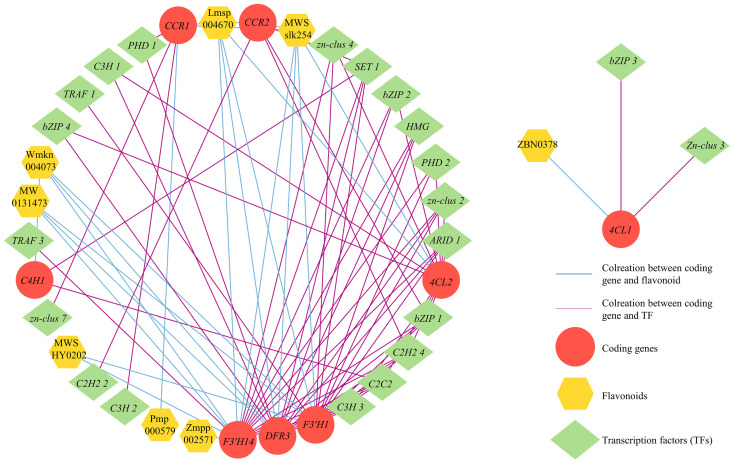
Transcriptional metabolic regulatory network associated with flavonoid synthesis in the fruiting body of *S. rugosoannulata*.

A correlation analysis between DEGs and DAFs in cluster 8 was performed to explore the connection between flavonoid metabolites and genes in *S. rugosoannulata* and to identify candidate TFs for regulating flavonoid biosynthesis. The analysis revealed that eight structural genes (*C4H1*, *4CL1*, *4CL2*, *F3*′*H1*, *F3*′*H14*, *DFR3*, *CCR1*, and *CCR2*) were highly correlated with eight flavonoid compounds (|r > 0.9|, *p* < 0.05, Figure 8; Table S8). Interestingly, *C4H1* and *4CL1*, which serve as the initiation regulatory genes for flavonoid biosynthesis, showed strong associations with two DAFs and eleven TFs and one DAF and two TFs, respectively. Compared to the upstream pathway, the downstream pathway exhibited a greater association with DAFs and TFs. *F3*′*H* demonstrated the highest number of associations with DAFs and TFs, and 5 DAFs and eleven TFs associated with *F3*′*H1*, and six DAFs and twelve TFs associated with *F3*′*H14*, respectively. *DFR3* was associated with four DAFs and twelve TFs. Furthermore, *CCR1* and *CCR2* were jointly associated with the same two DAFs and with three and four TFs, respectively. These results highlighted the crucial role of eight structural genes in regulating flavonoid biosynthesis in the fruiting body of *S. rugosoannulata*. Meanwhile, certain specific TFs, such as *C3H*, *C2H2*, *C2C2*, and *bZIP*, exhibited significant and positive correlations with the eight structural genes, indicating their potential involvement in regulating the expression of related structural genes and contributing to the differential accumulation of flavonoids.

### 3.6. Verification of Transcriptomics Data

**Figure 9:**
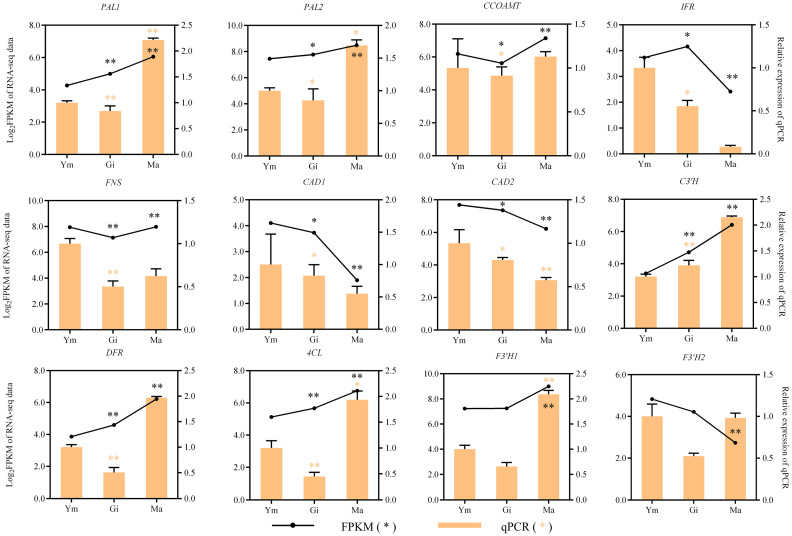
The expression patterns of genes involved in flavonoid biosynthesis were confirmed, with significant *p*-values of <0.05 (*) and <0.01 (**).

To verify the transcriptome data, 12 typical genes involved in flavonoid biosynthesis were chosen for expression analysis in Ym, Gi, and Ma by conducting qRT-PCR. The relative expression patterns of the majority of the candidate genes, as indicated in Figure 9, closely mirrored those observed in the transcriptome data obtained via Illumina sequencing. This congruence in expression patterns provides evidence supporting the validity and reliability of the transcriptome data.

## 4. Discussion

### 4.1. Presence of Genes Related to Flavonoid Biosynthesis in the Fruiting Body of S. rugosoannulata

Flavonoids, a diverse family of natural compounds widely distributed in plants, play crucial roles in plant growth, development, and stress adaptation [35]. Despite the abundance of secondary metabolites produced by mushrooms, whether they can synthesize flavonoids has been a subject of debate [36,37]. Recent advancements in metabolomics and transcriptomics have provided powerful tools for unraveling the mechanisms of secondary metabolite synthesis.

We initially extracted and quantified total flavonoids in *S. rugosoannulata* fruiting bodies at different stages of development. Our results revealed the consistent presence of flavonoids, with their concentration gradually increasing as the fruiting body matured. To further investigate the specific classes of flavonoids and the mechanisms involved in their synthesis in mushrooms, we subjected *S. rugosoannulata* fruiting bodies at different stages to UPLC-MS/MS and RNA-seq analysis.

We detected a total of 53 flavonoid metabolites, classified into eight categories based on flavonoid types. Notably, 33 of these flavonoids exhibited significant changes in abundance across different developmental stages. However, subsequent alignment of the transcriptome data to the *S. rugosoannulata* reference genome sequence only identified eight genes annotated to the flavonoid biosynthesis pathway (all associated with the phenylpropane biosynthesis pathway—two *PAL*, two *4CL*, and four *CCOAMT*). This discrepancy with the flavonoid metabolite results prompted us to undertake a non-participating transcriptome analysis to reconcile these inconsistencies.

Through de novo transcriptome assembly and analysis, we identified 59 unigenes associated with flavonoid biosynthesis. These unigenes were involved in pathways of phenylpropanoid biosynthesis (ko00940), flavonoid biosynthesis (ko00941), isoflavonoid biosynthesis (ko00943), and flavone and flavonol biosynthesis (ko00944), which aligned with the findings of the flavonoid metabolite analysis. Furthermore, the metabolome results indicated that flavones and flavonols made significant contributions to the fruiting body of *S. rugosoannulata* during all three development stages (44, 83.02%). This observation was further corroborated by the nonparticipating transcriptome analysis, which revealed the highest number of genes annotated to the flavonoid biosynthesis and the flavone and flavonol biosynthesis pathway (39, 66.10%). These findings strongly suggest that the fruiting body of *S. rugosoannulata* does contain genes dedicated to flavonoid synthesis.

To obtain high-quality genomes of mushrooms, researchers typically select monokaryotic strains in the asexual phase. This preference stems from several challenges associated with the heterothallic sexual reproduction stage and outcrossing during cultivation, including chromosome doubling and the complex reproductive system of mushrooms [38,39,40,41]. Selecting dikaryotic mycelium or fruiting bodies with reproductive abilities for genome sequencing has been observed to lead to high sequence heterozygosity, an inflated genome size, and unsatisfactory assembly effects [42,43,44]. This phenomenon is exemplified in mushroom species like *Leucocalocybe mongolica* [41], *Sanghuangporus baumii* [45], and *Pleurotus tuoliensis* [46], which have yielded high-quality genomes when sequenced and assembled with monokaryotic strains. Conversely, genome sequencing from dikaryotic mycelium or fruiting bodies of mushrooms like *Dictyophora indusiate* [42], *Russula griseocarnosa* [43], and *Agrocybe cylindracea* [44] has shown high sequence heterozygosity (N50 < 1 Mb), larger-than-actual genome sizes, and unsatisfactory assembly effects.

Fruiting bodies, multicellular structures produced by filamentous ascomycete fungi or basidiomycetes, play a crucial role in the reproduction and dispersal of sexual spores [47]. Their formation involves the differentiation of specific cells, a complex process characterized by coordinated patterns of gene expression in time and space [48]. Interestingly, mycelium development into fruiting bodies relies on the activation of specific genes in non-reproductive hyphae. These genes, primarily active at the transcriptional level, are less involved in the fundamental cellular functions and mechanisms of fungi [15,49]. Additionally, studies have shown that secondary metabolic gene clusters in fungi are often transcriptionally silenced under typical laboratory culture conditions [50,51]. For example, genome-wide microarray analyses of *Fusarium graminearum* fruiting body differentiation revealed that 12% of the transcripts were specific to the reproductive growth stage [52]. Similarly, LM/RNA-seq analysis of young fruiting bodies in *Sordaria macrospora* indicated that their overall gene expression resembled vegetative mycelium more than non-reproductive mycelium (protoperithecia) [53]. Further, 5′-Serial analysis of gene expression in *Coprinopsis cinerea* demonstrated a significant transcriptome turnover during the transition from mycelium to primordium [54].

In conclusion, we speculate that for fungi like *S. rugosoannulata* with reproductive capabilities, some genes inactive in non-reproductive mycelia become activated during fruiting body formation, alongside the activation of secondary metabolic gene clusters.

### 4.2. Key Regulatory Genes for Flavonoid Synthesis in the Fruity Body of S. rugosoannulata

Building upon our previous analysis, we utilized a non-participating transcriptome analysis approach to explore the transcriptome data comprehensively. After reconstructing the flavonoid biosynthesis pathway, we initially identified eight structural genes (*PAL1*, *C4H1*, *4CL2*, *F3*′*H10*, *F3*′*H11*, *F3*′*H14*, *DFR1*, and *DFR3*) that were consistently and significantly upregulated throughout the three growth phases of the *S. rugosoannulata* fruiting body. Subsequently, we constructed a correlation network between the transcriptome and metabolome data, revealing eight structural genes (*C4H1*, *4CL1*, *4CL2*, *F3*′*H1*, *F3*′*H14*, *DFR3*, *CCR1*, and *CCR2*) and 21 transcription factors (TFs) exhibiting strong correlations with flavonoid synthesis. This strongly suggests that these structural genes and TFs play crucial roles in the cumulative synthesis of flavonoids during the development of *S. rugosoannulata* fruiting bodies.

**Figure 10:**
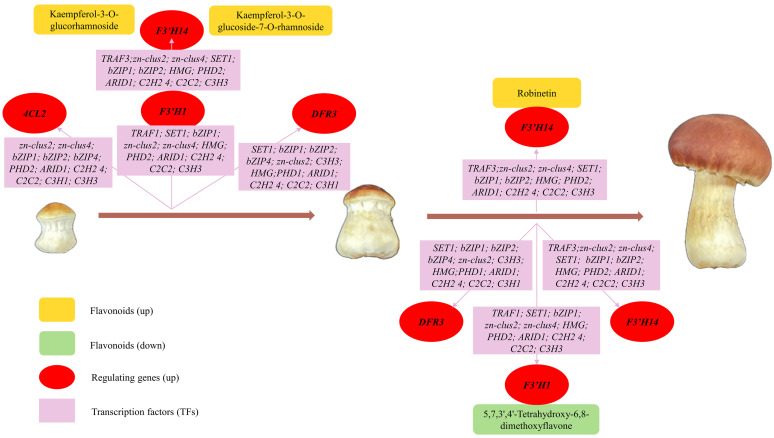
Changes in flavonoid content and their regulating structural genes and TFs during fruiting body development (Ym, Gi, and Ma stages).

As illustrated in Figure 10, the cumulative contents of kaempferol-3-O-glucorhamnoside [55,56], kaempferol-3-O-glucoside-7-O-rhamnoside [57], and robinetin [58] increased progressively. These three flavonoids with promising antioxidant, anti-inflammatory, anti-bacterial and anti-thrombotic effects and their increased content in the fruiting body of *S. rugosoannulata* may contribute significantly to its potential pharmaceutical value. Notably, kaempferol-3-O-glucorhamnoside and kaempferol-3-O-glucoside-7-O-rhamnoside were co-regulated by *4CL2*, *F3*′*H1*, *F3*′*H14*, and *DFR3*, with *F3*′*H14* playing a more pronounced role in robinetin synthesis. Conversely, the anti-inflammatory compound 5,7,3′,4′-tetrahydroxy-6,8-dimethoxyflavone exhibited a slight decrease in the late growth stage [59], possibly due to negative regulation by *F3*′*H1*, *F3*′*H14*, and *DFR3*. This observation highlights the dominant roles of *F3*′*H1*, *F3*′*H14*, and *DFR3* in overall flavonoid synthesis within the *S. rugosoannulata* fruiting body. Additionally, it suggests the possibility of tailoring mushroom harvest timing to optimize specific flavonoid content according to our needs.

Previous studies have annotated the presence of four genes related to flavonoid synthesis in *S. baumii* (*PAL*, *4CL*, *CHI*, and *IFR*) [13] and three in *Auricularia cornea* (*PAL*, *PPO*, and *CHI*) [12]. These findings suggest that *PAL*, *4CL*, and *CHI* play crucial roles in initiating flavonoid biosynthesis in both mycelium and fruiting bodies. However, a lack of research on flavonoid biosynthesis in mushroom fruiting bodies has limited our understanding of genes like *F3*′*H* and *DFR*.

Research on plant flavonoid biosynthesis has identified and characterized the roles of *F3*′*H* and *DFR* in regulating flavonoid production. For example, the importance of *F3*′*H* in the accumulation of flavonoids in various plant species, including apples (*malus domestica borkh*) [60], *Camellia nitidissima* Chi [61], *Fagopyrum tataricum* Gaertn [62], and *Dendrobium huoshanense* [63]. In purple Chinese cabbage, *F3*′*H* has been identified as a key enzyme determining the characteristics of the flavonoid metabolic profile. Interestingly, it also exhibits significantly higher catalytic efficiency for kaempferol than dihydrokaempferol (DHK), suggesting kaempferol as the preferred substrate [64]. This potential preference in *S. rugosoannulata* may explain the higher levels of flavone and flavonol metabolites observed, but further investigation is needed to confirm this conjecture.

Similarly, studies have shown that transcriptional regulation of flavonoid genes, particularly *DFR* genes, plays a role in regulating flavonoid biosynthesis in various plant species like Chrysanthemum [65], Pepper [66], and Sorghum [67]. However, the regulatory roles of these genes in fungal fruiting bodies remain unexplored.

In conclusion, exploring flavonoid metabolic profiles in fungal fruiting bodies, investigating flavonoid-related genes, and elucidating the regulatory mechanisms of associated transcription factors hold significant value for further research. This underlines the fact that the study of flavonoids in fungal fruiting bodies is still in its initial stages and has immense potential for future investigation.

## Figures and Tables

**Table 1:** Top 10 DAFs with |log2 (fold change)| in each sample comparison.

Index	Compounds	Class	Log2 (Fold Change)	*p*-Value	Type
Ym vs. Gi					
ZBN0290	Acacetin-7-O-glucoside	Flavones	2.54	1.29 × 10^−2^	up
MW0139530	Quercetin 3-xylosyl-(1->6)-glucoside	Flavones	2.20	1.69 × 10^−5^	up
HJN091	Prunetin-4′-O-glucoside	Isoflavones	1.60	3.16 × 10^−3^	up
Lmsp004166	Quercetin-3-O-glucoside-7-O-rhamnoside	Flavonols	−1.73	5.34 × 10^−2^	down
Lmjp002461	Quercetin-3-O-neohesperidoside	Flavonols	−1.73	5.34 × 10^−2^	down
MWSHY0067	Quercetin-3-O-rutinoside	Flavonols	−2.05	1.13 × 10^−1^	down
Zbsp004301	Quercetin-7-O-rutinoside	Flavonols	−2.05	1.13 × 10^−1^	down
pmn001583	Quercetin-3-O-robinobioside	Flavonols	−2.33	1.85 × 10^−2^	down
MWSHY0016	Luteolin-6-C-glucoside	Flavones	−2.78	5.86 × 10^−3^	down
MWSHY0016	Luteolin-8-C-glucoside	Flavones	−3.75	5.76 × 10^−2^	down
Gi vs. Ma					
mws0036	Hesperetin-7-O-rutinoside	Flavanones	1.15	2.22 × 10^0^	up
Zmjp005293	Robinson-7-O-Neohesperidin	Flavonols	2.52	5.75 × 10^0^	up
Zmpp002571	Robinetin	Flavonols	2.17	4.15 × 10^0^	up
MW0139530	Quercetin 3-xylosyl-(1->6)-glucoside	Flavones	−1.47	3.16 × 10^−1^	down
ZBN0290	Acacetin-7-O-glucoside	Flavones	−1.48	3.59 × 10^−1^	down
ZBN0180	Kaempferol-3-O-(4″-O-acetyl)rhamnoside	Flavonols	−2.07	2.38 × 10^−1^	down
MW013147	Tetrahydroxy-6,8-dimethoxyflavone	Flavones	−1.02	4.95 × 10^−1^	down
Zmyn003693	6-Hydroxy-2′-methoxyflavone	Flavones	−1.01	4.97 × 10^−1^	down
Ym vs. Ma					
Zmpp002571	Robinetin	Flavonols	2.36	2.91 × 10^−5^	up
MWSslk254	Kaempferol-3-O-glucorhamnoside	Flavonols	1.83	8.06 × 10^−4^	up
Lmsp004670	Kaempferol-3-O-glucoside-7-O-rhamnoside	Flavonols	1.83	8.06 × 10^−4^	up
Lmjp003655	6-C-MethylKaempferol-3-glucoside	Flavones	1.36	3.78 × 10^−2^	up
mws1290	Kaempferol-3-O-(6″-p-Coumaroyl)glucoside	Flavonols	1.18	9.72 × 10^−2^	up
ZBN0378	Afzelechin-(4α→8)-epiafzelechin	Flavonols	−1.29	4.12 × 10^−2^	down
MWSHY0067	Quercetin-3-O-rutinoside	Flavonols	−1.55	1.46 × 10^−1^	down
Zbsp004301	Quercetin-7-O-rutinoside	Flavonols	−1.55	1.46 × 10^−1^	down
MWSHY0016	Luteolin-6-C-glucoside	Flavones	−2.32	1.58 × 10^−2^	down
mws1299	Luteolin-8-C-glucoside	Flavones	−2.98	4.53 × 10^−2^	down

Note: If the number of DAFs is less than 10, all are displayed.

**Table 2:** The main flavonoid-related pathways associated with *S. rugosoannulata*.

KEGG Pathway	Ym vs. Gi			Gi vs. Ma			Ym vs. Ma		
	Rich Factor	*p*-Value	Count	Rich Factor	*p*-Value	Count	Rich Factor	*p*-Value	Count
Phenylpropanoid biosynthesis	1.68	5.58 × 10^−2^	12	1.46	1.17 × 10^−2^	37	1.49	4.13 × 10^−3^	43
Flavonoid biosynthesis	1.59	2.10 × 10^−2^	12	1.78	2.09 × 10^−4^	39	1.57	2.40 × 10^−3^	39
Isoflavonoid biosynthesis	2.67	2.51 × 10^−2^	6	1.00	5.61 × 10^−1^	8	0.88	7.07 × 10^−1^	8
Flavone and flavonol biosynthesis	1.80	1.44 × 10^−1^	5	2.32	6.50 × 10^−5^	23	1.88	2.63 × 10^−3^	21

**Table 3:** Top 10 DEGs with |log2 (fold change)| involved in flavonoid-related pathway among various sample pairs.

Gene ID	Gene Name	Protein Function	EC Number	Log2 (Fold Change)	*p*-Value	Type
Ym vs. Ma						
Cluster-11040.14	*4CL1*	hypothetical protein	EC:6.2.1.12	1.31	3.57 × 10^−3^	up
Cluster-6482.6	*C3*′*H4*	trimethyltridecatetraene synthase-like	EC:1.14.14.96	1.23	4.44 × 10^−3^	up
Cluster-10276.0	*CCR2*	hypothetical protein	EC:1.2.1.44	7.14	3.09 × 10^−7^	up
Cluster-5081.0	*DFR3*	hypothetical protein	EC:1.1.1.219 1.1.1.234	2.95	5.90 × 10^−8^	up
Cluster-8590.8	*F3*′*H14*	cytochrome P450	EC:1.14.14.82	1.83	9.07 × 10^−16^	up
Cluster-1699.0	*F3H*	flavanone 3-hydroxylase	EC:1.14.11.9	−2.56	1.04 × 10^−16^	down
Gi vs. Ma						
Cluster-6482.6	*C3*′*H4*	trimethyltridecatetraene synthase-like	EC:1.14.14.96	1.89	6.66 × 10^−89^	up
Cluster-10227.0	*CCOAMT*	hypothetical protein L1887_45734	EC:2.1.1.104	1.43	3.48 × 10^−26^	up
Cluster-10183.3	*DFR1*	hypothetical protein L1887_49712	EC:1.1.1.219 1.1.1.234	1.83	5.69 × 10^−42^	up
Cluster-8045.2	*F3*′*H9*	hypothetical protein FH972_026701	EC:1.14.14.82	1.70	1.63 × 10^−3^	up
Cluster-8045.6	*F3*′*H10*	cytochrome P450	EC:1.14.14.82	1.93	3.35 × 10^−183^	up
Cluster-8590.8	*F3*′*H14*	cytochrome P450	EC:1.14.14.82	1.30	9.12 × 10^−19^	up
Cluster-8459.0	*CAD2*	hypothetical protein L1887_42355	EC:1.1.1.195	−1.66	9.76 × 10^−29^	down
Cluster-10281.0	*DFR2*	hypothetical protein L1887_49712	EC:1.1.1.219 1.1.1.234	−3.28	5.59 × 10^−42^	down
Cluster-4637.0	*IFS3*	hypothetical protein FH972_026701	EC:1.14.14.90 1.14.14.89	−6.41	1.37 × 10^−68^	down
Cluster-4637.4	*IFS4*	hypothetical protein FH972_026701	EC:1.14.14.90 1.14.14.89	−1.54	4.49 × 10^−16^	down
Ym vs. Ma						
Cluster-6482.6	*C3*′*H4*	trimethyltridecatetraene synthase-like	EC:1.14.14.96	3.12	3.12 × 10^−171^	up
Cluster-10276.0	*CCR2*	hypothetical protein	EC:1.2.1.44	7.50	2.75 × 10^−9^	up
Cluster-5081.0	*DFR3*	hypothetical protein	EC:1.1.1.219 1.1.1.234	4.01	3.51 × 10^−15^	up
Cluster-8590.8	*F3*′*H14*	cytochrome P450	EC:1.14.14.82	3.12	2.79 × 10^−62^	up
Cluster-3679.0	*CHS1*	chalcone synthase 2	EC:2.3.1.74	−2.93	5.99 × 10^−3^	down
Cluster-10281.0	*DFR2*	hypothetical protein L1887_49712	EC:1.1.1.219 1.1.1.234	−2.52	3.77 × 10^−21^	down
Cluster-4637.0	*IFS3*	hypothetical protein FH972_026701	EC:1.14.14.90 1.14.14.89	−6.01	3.03 × 10^−56^	down
Cluster-5642.0	*LAR*	leucoanthocyanidin reductase-like	EC:1.17.1.3	−2.64	4.07 × 10^−3^	down
Cluster-3443.0	*peroxidase1*	peroxidase 42-like	EC:1.11.1.7	−2.87	6.92 × 10^−3^	down
Cluster-7458.0	*peroxidase2*	hypothetical protein HYC85_021248	EC:1.11.1.7	−3.24	8.46 × 10^−3^	down

Note: If the number of DEGs is less than 10, all are displayed.

## Data Availability

The sequencing data mentioned in this research can be downloaded from NCBI (www.ncbi.nlm.nih.gov, accessed on 23 January 2024). Number of bioproject: PRJNA1066961. The remaining data can be found in the article or Supplementary Materials.

## Supplementary Materials

The following supporting information can be downloaded at: https://www.mdpi.com/article/10.3390/jof10040254/s1, Table S1: List of primers used in this study; Table S2: Flavonoids from the fruiting body of *S. rugosoannulata*; Table S3: Thirty-three DAFs between different pairs of samples; Table S4: Summary information on RNA-seq for *S. rugosoannulata*; Table S5: In total, 6726 DEGs can be seen between different pairs of samples; Table S6: In total, 59 unigenes involved in the flavonoid-related pathway can be seen; Table S7: Transcription factors in cluster 8; Figure S1: Diagram of the total ion current for sample quality spectrum analysis; Figure S2: Parameters and permutation test for the OPLS-DA model.
