# Supplementary material for: Integrative Analysis of Transcriptome and Metabolome Sheds Light on Flavonoid Biosynthesis in the Fruiting Body of Stropharia rugosoannulata
Source: J Fungi (Basel). 2024 Mar 27;10(4):254. doi: 10.3390/jof10040254 (PMC11051051; doi:10.3390/jof10040254)
Supplement: Supplementary file 1 [file jof-10-00254-s001.zip › Figure S1.pdf]

Figure S1. Diagram of the total ion current for sample quality spectrum analysis.

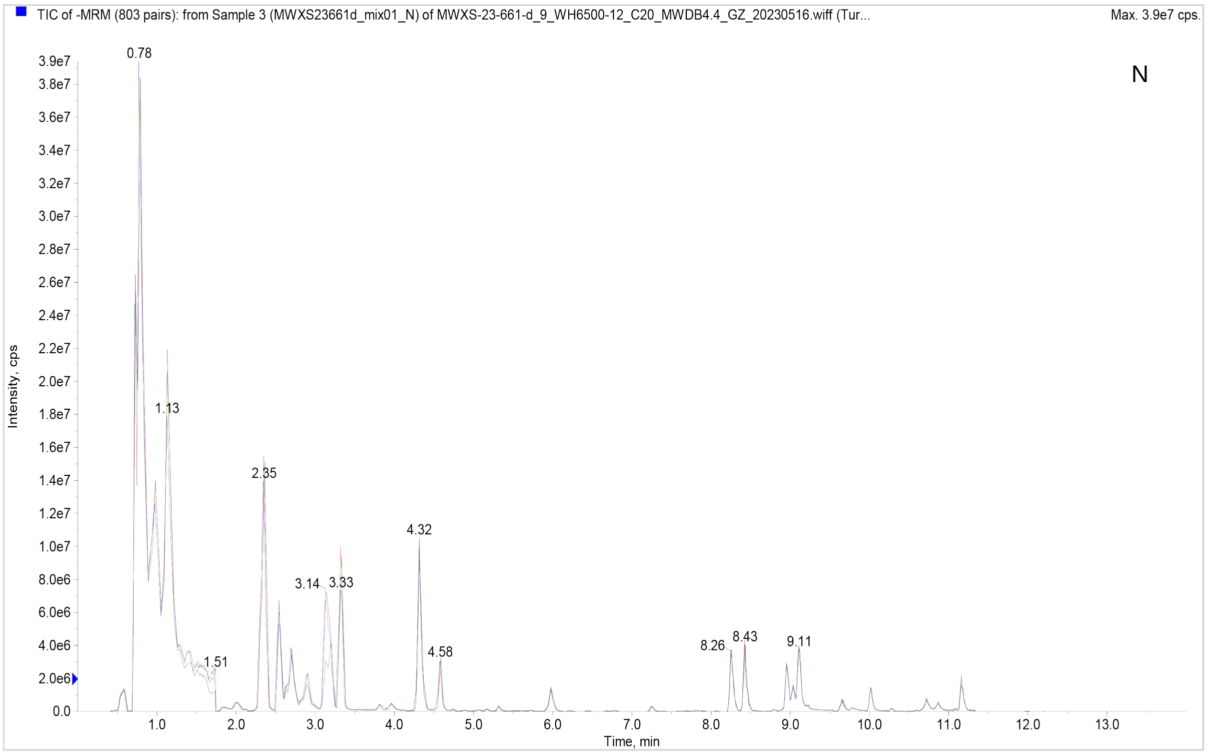

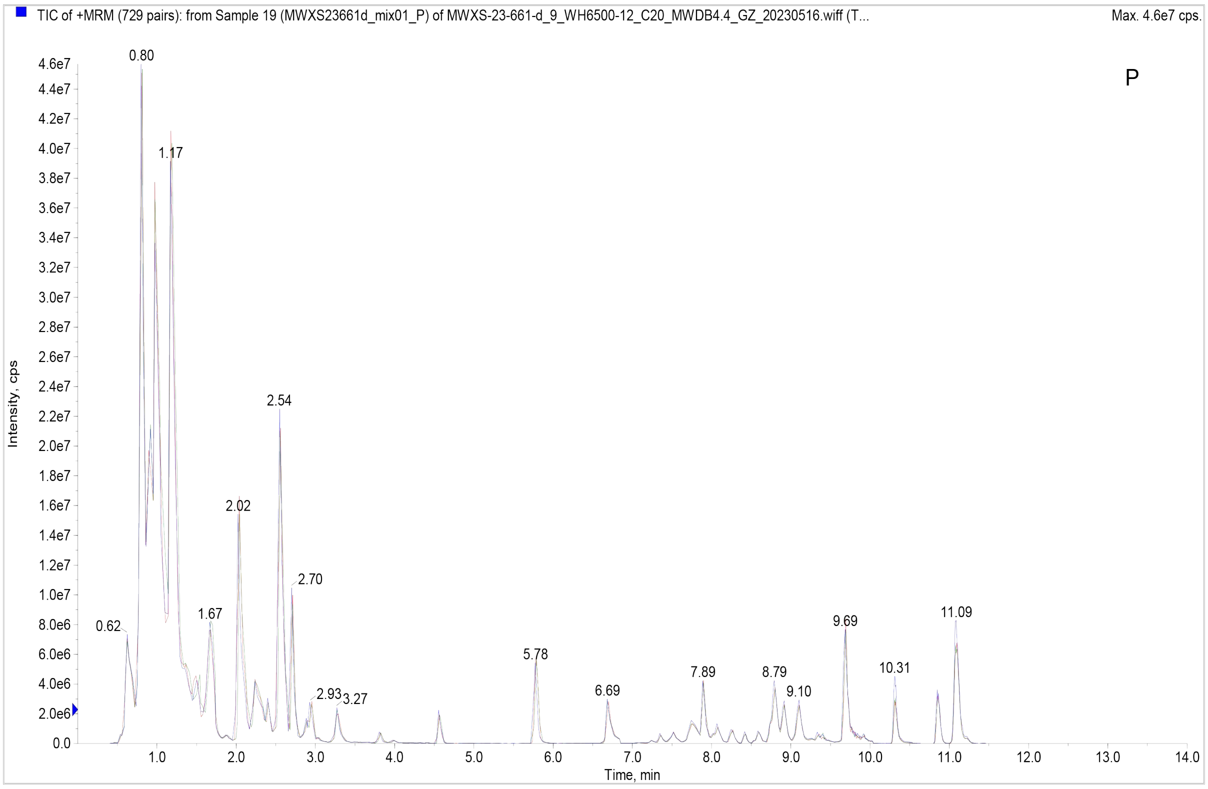

Note: The x-coordinate was the retention time (min) of the metabolite detection and the y-coordinate was the ion current intensity (cps) of a metabolite ion detection.
