# Supplementary figures and images for: Integrative Analysis of Transcriptome and Metabolome Sheds Light on Flavonoid Biosynthesis in the Fruiting Body of Stropharia rugosoannulata
Source: J Fungi (Basel). 2024 Mar 27;10(4):254. doi: 10.3390/jof10040254 (PMC11051051; doi:10.3390/jof10040254)

**Figure S2.** Parameters and permutation test for the OPLS-DA model.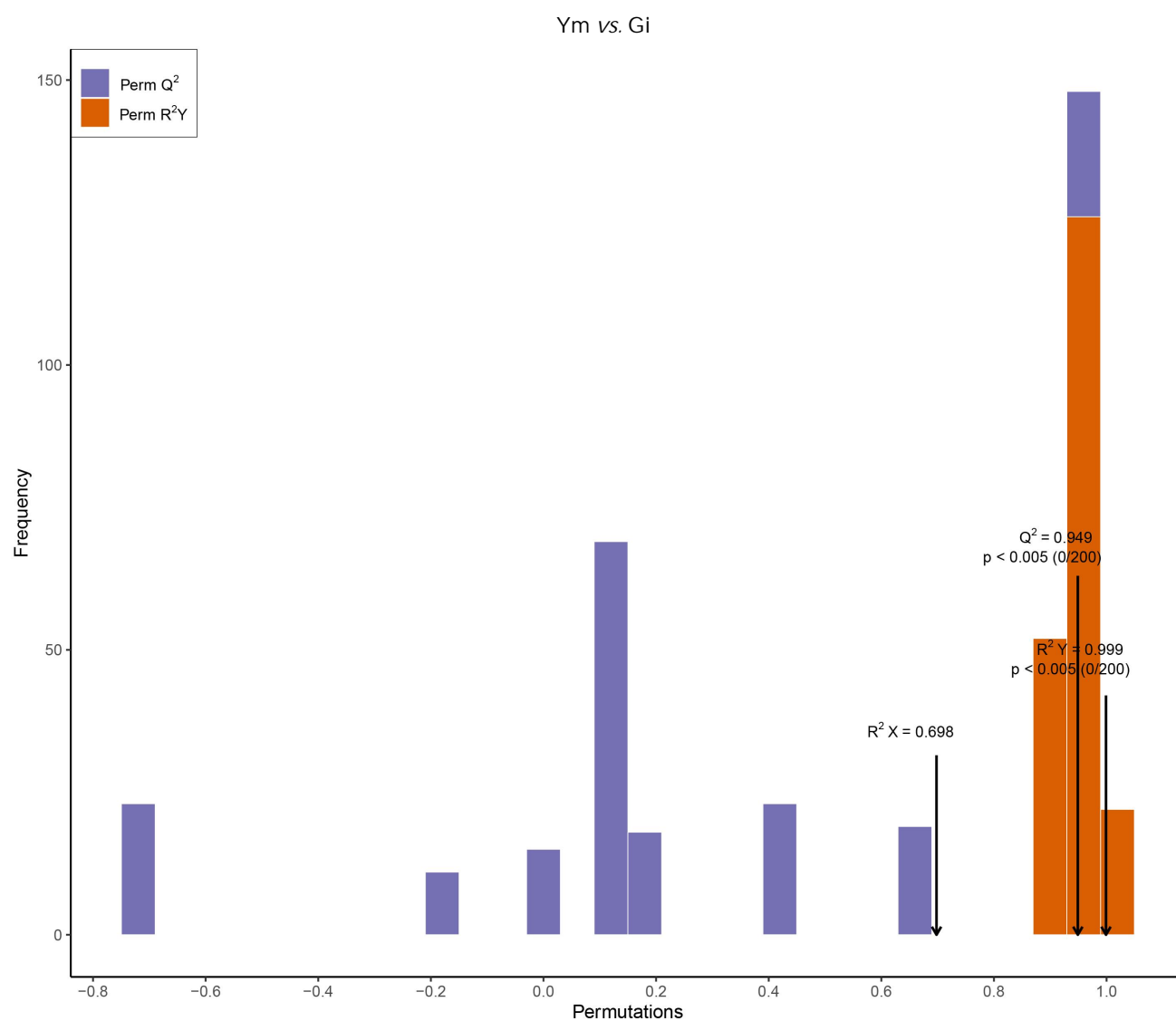

Gi vs. Ma

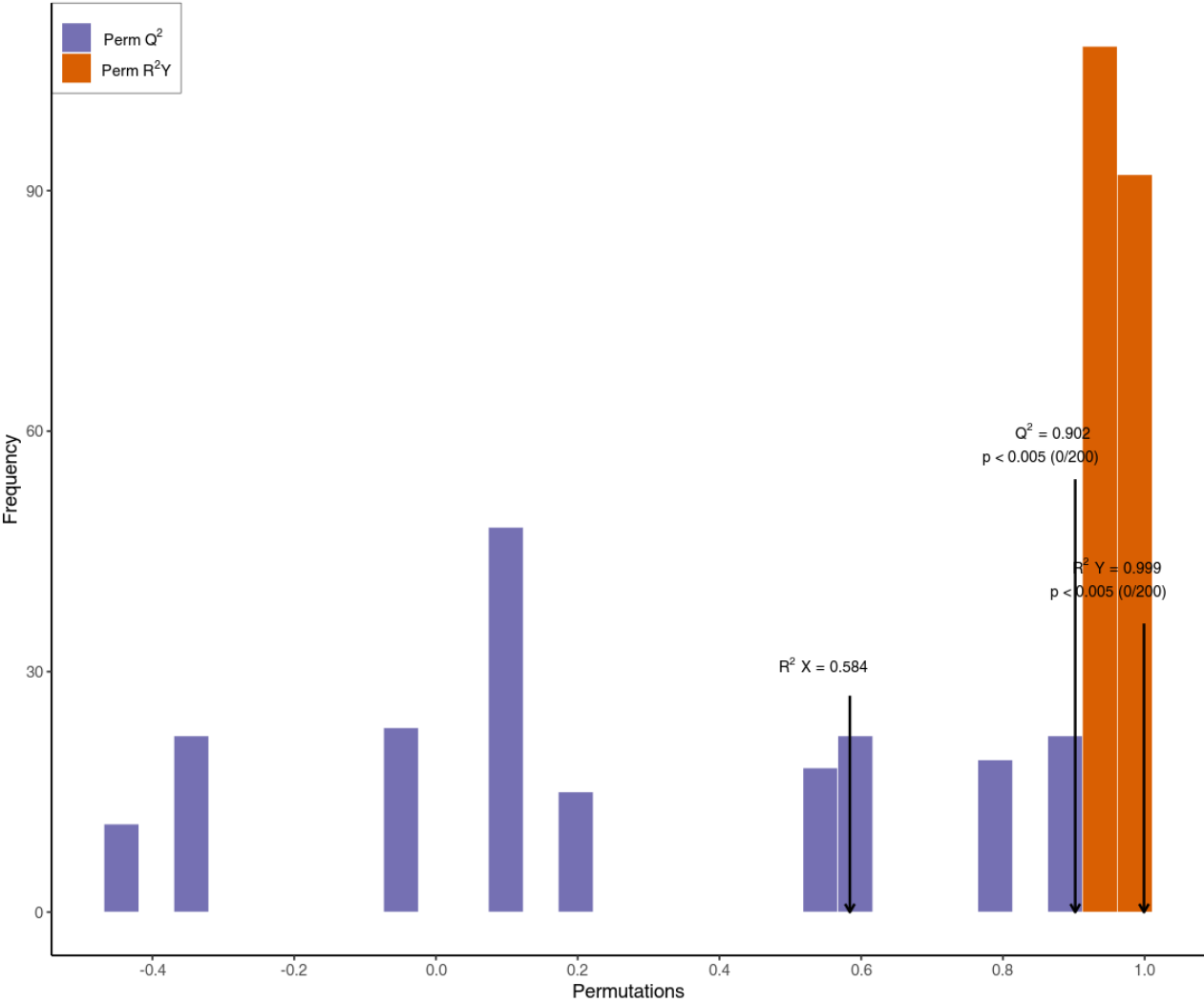

Ym vs. Ma

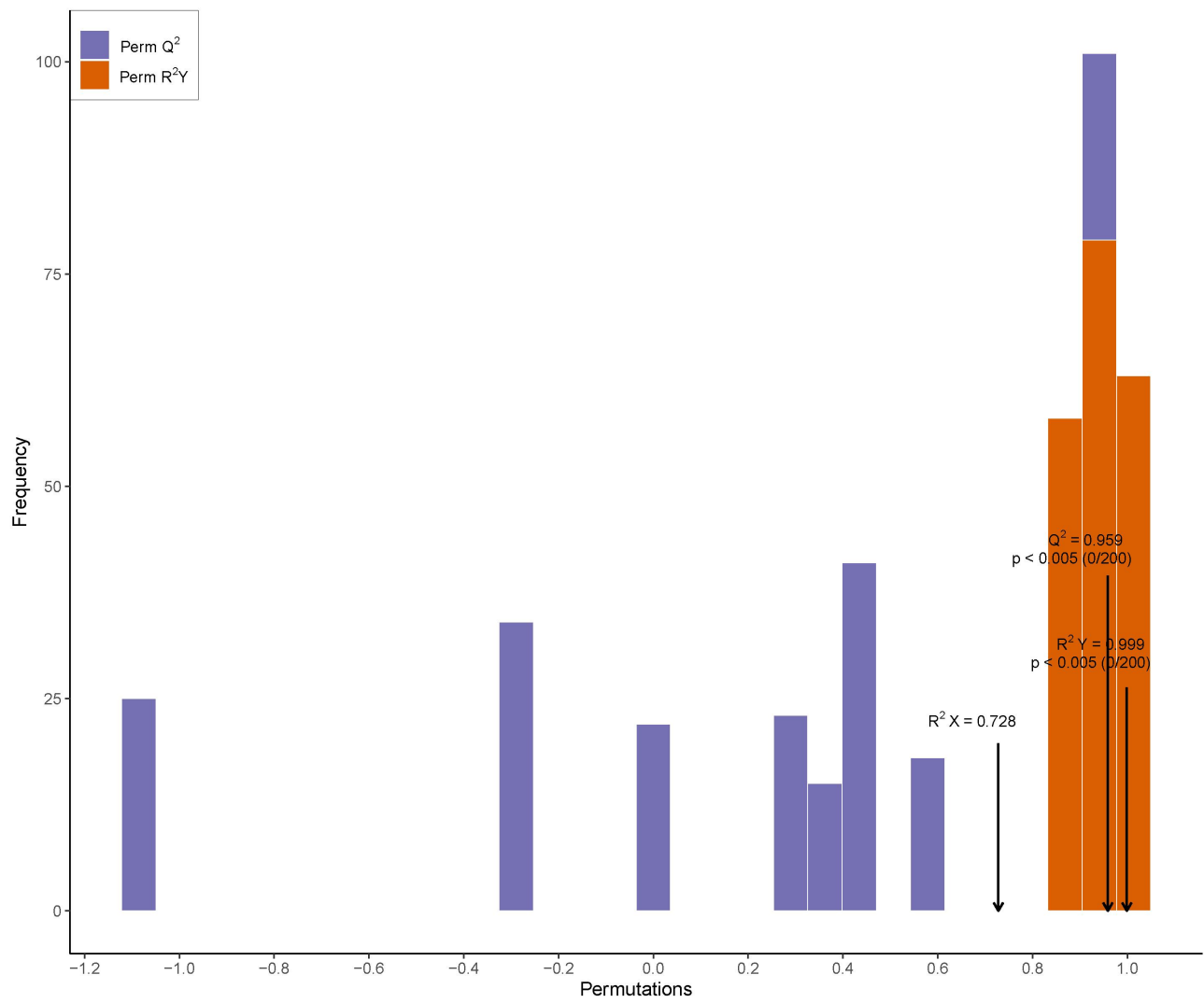

Supplement: Supplementary file 1 [file jof-10-00254-s001.zip › Figure S2.pdf]
